# Radiation Exposure of Cardiac Conduction Nodes During Breast Proton Therapy

**DOI:** 10.14338/IJPT-22-00038.1

**Published:** 2023-03-09

**Authors:** Pierre Loap, Farid Goudjil, Vincent Servois, Krassen Kirov, Alain Fourquet, Youlia Kirova

**Affiliations:** 1 Department of Radiation Oncology, Institut Curie, Paris, France; 2 Department of Radiology, Institut Curie, Paris, France; 3 Department of Anesthesiology, Institut Curie, Paris, France

**Keywords:** cardiac substructure, IMRT, proton therapy, cardiotoxicity

## Abstract

**Purpose:**

The exposition of cardiac conduction system during breast radiation therapy has never been studied, despite the increasing use of intensity-modulated radiation therapy, which exposes larger volume to low-dose bath. We evaluated conduction node exposure during breast irradiation with volumetric modulated arc therapy and estimated the potential dosimetric benefit with intensity-modulated proton therapy.

**Materials and Methods:**

Atrioventricular (AVN) and sinoatrial (SAN) nodes were retrospectively delineated according to published guidelines on the simulation computed tomography scans of 12 breast cancer patients having undergone conserving surgery and adjuvant locoregional volumetric modulated arc therapy. Intensity-modulated proton therapy treatment was replanned on the simulation computed tomography scans for all breast cancer patients. Mean and maximum doses delivered to the SAN and the AVN were retrieved and compared. Correlation coefficients were calculated between doses to the SAN or the AVN and the whole heart.

**Results:**

Average mean doses delivered to the SAN and AVN were 2.8 and 2.3 Gy, respectively, for left-sided irradiation and 9.6 and 3.6 Gy, respectively, for right-sided irradiation. Average maximum doses to the SAN and AVN were 3.5 Gy and 2.8 Gy, respectively, for left-sided irradiation and 13.1 and 4.6 Gy, respectively, for right-sided irradiation. Intensity-modulated proton therapy significantly reduced mean and maximum doses to the SAN and AVN. Correlations between doses to the SAN or AVN and whole heart were usually significant.

**Conclusion:**

SAN and AVN can be substantially exposed during breast volumetric modulated arc therapy, especially for right-sided irradiation. Cardiotoxicity studies evaluating conduction node exposure might define dose constraints and criteria for additional cardiac-sparing techniques, such as respiratory techniques or proton therapy, which could benefit patients with underlying rhythmic or conduction disorders.

## Introduction

Adjuvant breast radiation therapy is associated with increased locoregional control and patient-specific survival [1]. Irradiation techniques have evolved and currently can efficiently spare the heart and its critical substructures, such as the coronary arteries, even in the case of unfavorable anatomy [2, 3]. Such techniques include rotational intensity-modulated radiation therapy (IMRT) or proton therapy. The heart is a complex organ at risk, and the multiplicity of its substructures explains the vast range of described radiation-induced cardiotoxicity types [4]. Arrhythmias and conduction disorders are described as radiation-induced complications of thoracic irradiations [5]. With the development of cardiac conduction system delineation atlases [6], evaluating the relationship between conduction disorders or arrhythmias and conduction substructure exposure has become possible. For lung cancer irradiation, it has been demonstrated that the mean dose to the sinoatrial node (SAN) was correlated with atrial fibrillation and mortality [7].

However, such a dose-toxicity relationship is yet to be demonstrated for breast cancer radiation therapy. For breast radiation therapy, IMRT is increasingly used when the coronary risk is deemed important [8], at the expense of an increased low-dose bath, especially for its rotational variants, such as volumetric modulated arc therapy (VMAT) [9]. In this context, the dosimetric consequences on the cardiac conduction system, which is mainly localized on the posterior part of the heart, have never been investigated. The SAN is lateralized on the right side of the heart, and its radiation exposure is very likely to be influenced laterality by breast cancer. Proton therapy is increasingly evaluated for breast cancer treatment [10, 11] based on its ability to spare coronary arteries; its dosimetric benefit for cardiac conduction sparing is currently unknown but may justify its use for patients with underlying arrhythmic or conduction diseases.

This study evaluated conduction node exposure during breast irradiation using VMAT. It also compared the dosimetric performances of intensity-modulated proton therapy (IMPT) and VMAT at this level.

## Materials and Methods

### Population

This study was conducted in the Department of Radiation Oncology (Institut Curie, Paris, France). Seven left-sided and 5 right-sided breast cancer patients treated between December 2016 and October 2019 were randomly selected from our institutional database. All included patients were treated for breast carcinoma by conserving surgery and subsequently irradiated with VMAT, which was validated by a quality control staff based on anatomic considerations (such as pectus excavatum) and dosimetric considerations (unacceptable doses to organs at risk with 3D techniques), and which target volumes included the whole breast with a boost and regional lymph nodes, including the internal mammary chain.

### VMAT Treatment and Proton Therapy Replanning

Patients were immobilized supine with both arms above the head. All simulation computed tomography (CT) scans were acquired with the same parameters (noncontrast and using 3-mm slices) and transferred to the Eclipse 8.9 treatment planning system (Varian Medical Systems) for VMAT planning. All patients had been treated with free-breathing, normofractionated VMAT in 28 fractions. Target volumes included the whole breast (51.8 Gy) with a boost (63 Gy) and Berg’s level II-IV, interpectoral, and internal mammary lymph nodes (50.4 Gy). Planned target volumes (PTV) were defined with a 5-mm expansion margin around clinical target volumes. A minimum of 95% of the prescription dose should be delivered to 95% of the PTV, and a maximum of 107% of the prescription dose could be delivered to 2% of the PTV.

IMPT was retrospectively replanned on the simulation CT scans for all patients, on the Eclipse proton treatment planning system, by medical physicists unaware of the initial VMAT treatment plans. Similarly, the prescribed doses were 51.8 GyRBE to the whole breast, 63 GyRBE to the tumor bed boost, and 50.4 GyRBE to Berg’s levels II through IV, interpectoral, and internal mammary lymph nodes. One single enface field was planned on an IBA C230 cyclotron with a nominal proton beam energy of 230 MeV and a 65-mm acrylic range shifter. PTV were defined with a 5-mm isotropic expansion margin around clinical target volumes. Optimization constraints were similar to those of the VMAT plans.

### Cardiac Conduction Node Delineation

The cardiac conduction system exposure evaluation followed a previously reported methodology described in a dosimetric study from our research group focusing on mediastinal lymphoma patients [12]. On the patients’ simulation CT scans, we retrospectively delineated the AVN and sinoatrial node SAN according to the published delineation atlas [6], and each contour was checked by a multidisciplinary team consisting of a radiologist and 2 radiation oncologists. The AVN corresponded to a 2-cm diameter sphere, tangent to the right atrium and centered at the height of the ascending aorta origin, while the AVN corresponds to a 2-cm diameter sphere centered at the intersection of the 4 cardiac cavities, 1 cm above the inferior extremity of the left atrium.

### Statistics

Mean and maximum doses delivered to the AVN and SAN were retrieved from the dose-volume histograms of the VMAT and IMPT plans and compared with Wilcoxon signed-rank tests. Pearson correlation coefficient matrices were calculated between doses to the AVN, SAN, and whole heart. Statistical analyses were performed using R 4.0.1 software.

## Results

**Figure 1.**
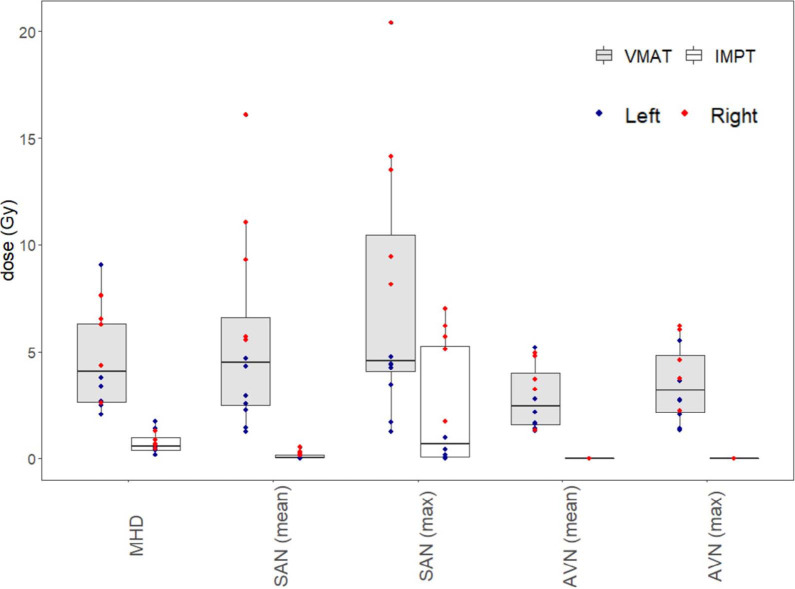
Cardiac conduction node exposure during locoregional breast irradiation with volumetric modulated arc therapy (VMAT) or intensity-modulated proton therapy (IMPT). Abbreviations: MHD, mean heart dose; SAN, sinoatrial node; AVN, atrioventricular node.

**Figure 2.**
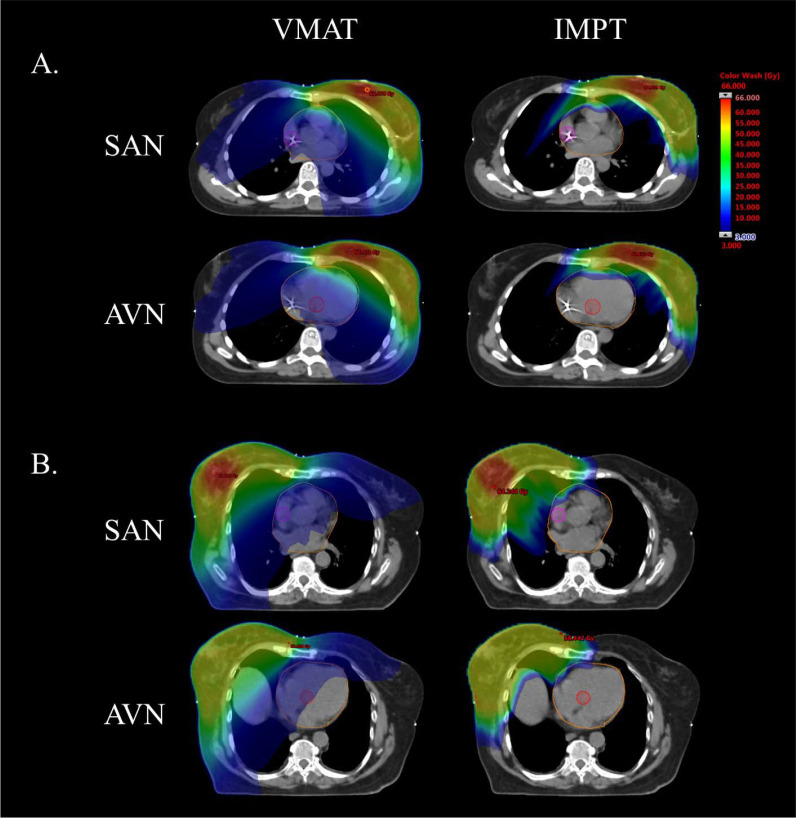
Conduction node exposure with volumetric modulated arc therapy (VMAT) and intensity-modulated proton therapy (IMPT). Sinoatrial node (SAN) is delineated in magenta, atrioventricular node (AVN) in red, and the heart in orange. (A) Left-sided breast cancer radiation therapy. (B) Right-sided breast cancer radiation therapy.

Average mean doses delivered to the SAN and AVN were 2.8 and 2.3 Gy, respectively, for left-sided irradiation and 9.6 and 3.6 Gy, respectively, for right-sided irradiation, with VMAT (**Figure 1**). Average maximum doses to the SAN and AVN were 3.5 and 2.8 Gy, respectively, for left-sided irradiation and 13.1 and 4.6 Gy, respectively, for right-sided irradiation, with VMAT. For left-sided irradiation, IMPT significantly reduced the mean dose to the SAN from 2.8 to 0.0 Gy and the AVN from 2.3 to 0 Gy, and the maximum dose to the SAN from 3.5 to 0.2 Gy and the AVN from 2.8 to 0 Gy (*P* < .01). For right-sided irradiation, IMPT significantly reduced mean dose to the SAN from 9.6 to 0.3 Gy and to the AVN from 3.6 to 0 Gy, and the maximum dose to the SAN from 13.1 to 5.2 Gy and the AVN from 4.6 to 0 Gy (*P* < .01). An example of conduction node contours on the left- and right-sided VMAT and IMPT plans is provided in **Figure 2**. Correlations were strong between mean heart dose (MHD) and AVN mean dose (*r* = .94, *P* < .001) or AVN maximum dose (*r* = .89, *P* < .01), and moderate between MHD and SAN mean dose (*r* = .60, *P* < .05) or SAN maximum dose (*r* = .54, *P* = .07).

## Discussion

This study quantified the radiation exposure of cardiac conduction nodes during breast radiation therapy using VMAT. VMAT is associated with an increased low-dose bath compared with classic 3D radiation therapy, which may overexpose posteriorly located cardiac substructures such as the SAN. The SAN seemed more exposed with right-sided VMAT than left-sided VMAT, which can be explained by the fact that the SAN is located on the right side of the heart. Consequently, risks of conduction disorders might be different according to the cancer laterality, and cardiac sparing techniques (such as deep inspiration breath hold or gating) might be beneficial for right-sided breast cancer patients with underlying rhythmic or conduction disorders for whom conduction node sparing should be theoretically recommended, despite lack of clear clinical evidence.

Until recently, the heart was frequently considered a homogeneous organ at risk, and the MHD was the most frequently evaluated dosimetric parameter [13]. MHD tended to correlate with mean and maximum doses delivered to the SAN and AVN with VMAT, which suggests that dosimetric parameters to the conduction nodes could be inferred from the MHD for breast VMAT. Recently published clinical trials have found that specific cardiac substructure dosimetric parameters correlated with clinical cardiac adverse events [14, 15]; in particular, atrial fibrillation and overall survival were statistically associated with SAN mean dose during lung radiation therapy and for non-small cell lung squamous cell carcinoma, a SAN mean dose constraint of 20 Gy was proposed. For breast cancer (for which such dose toxicity association is yet to be demonstrated), long-term and retrospective cardiotoxicity studies considering the cardiac conduction system and focusing on rhythmic disorders may similarly define such dose constraints [16]. An exploratory case-control epidemiologic study found a slightly higher risk of arrhythmia for right-sided breast cancer irradiation than left-sided irradiation [16], which might be related to a higher dose distribution to right-sided cardiac substructures, in particular the SAN; however, larger studies are needed to confirm this hypothesis. Currently, dose constraints to the conduction nodes, which may allow active avoidance with VMAT, are yet to be precisely defined for breast cancer radiation therapy [16, 17]. Non-coplanar VMAT planning with partial arcs may potentially lead to a better sparing of the conduction nodes compared with the standard coplanar approach used in this study [18]. Manual delineation of SAN and AVN is time-consuming, but cardiac auto-segmentation algorithms have shown promising results, especially when substructure sizes were large enough [19, 20] and implementation of SAN and AVN in auto-segmentable cardiac atlas has proven feasible [21].

The limits of this study, inherent to its dosimetric objective, include its size and retrospective nature. In addition, because the AVN and SAN were retrospectively contoured for this study, no active constraints were used to voluntarily avoid the cardiac conduction nodes when the VMAT treatment was initially planned. We only included patients with locoregional VMAT irradiation indication, including the internal mammary chain. This population represents a small proportion of all breast cancer patients frequently treated with IMRT. The youngest patients of this population, in particular those treated for left-sided breast cancers, are increasingly evaluated for breast proton therapy indication, which drastically reduces doses to critical cardiac substructures. Incidentally, we found that proton therapy delivers virtually no dose to cardiac conduction nodes and might suppress radiation-induced conduction and rhythmic disorder risk. As opposed to our study population, patients who do not require regional lymph node irradiation, including the internal mammary chain, may have a lower radiation exposure to the cardiac conduction nodes, in particular, cases of partial breast irradiation with brachytherapy or external radiation therapy, which significantly reduces doses delivered to the cardiac substructures [22].

Currently, most indications for breast proton therapy are based on toxicity models based on MHD and doses delivered to cardiac substructures (such as the left anterior coronary artery) on optimal photon radiation therapy plans [11, 23, 24]. Our findings suggest that patients with severe cardiac arrhythmia or conduction disorders might also represent potential candidates for breast proton therapy.

In conclusion, SAN and AVN can be substantially exposed during breast VMAT, especially for right-sided irradiation. Cardiotoxicity studies evaluating conduction node exposure might define dose constraints and criteria for additional cardiac sparing techniques, such as breathing control techniques or proton therapy, which delivers virtually no dose to cardiac conduction nodes and might benefit patients with underlying rhythmic or conduction disorders.
